# High Insulin Levels in KK-Ay Diabetic Mice Cause Increased Cortical Bone Mass and Impaired Trabecular Micro-Structure

**DOI:** 10.3390/ijms16048213

**Published:** 2015-04-13

**Authors:** Cen Fu, Xiaolin Zhang, Fei Ye, Jianhong Yang

**Affiliations:** 1 College of Life Science, University of Chinese Academy of Sciences, Beijing 100049, China; E-Mail: fucen2010@126.com; 2 Beijing Key Laboratory of New Drug Mechanisms and Pharmacological Evaluation Study, Institute of MateriaMedica, Chinese Academy of Medical Sciences, Beijing 100050, China; E-Mail: zhangxiaolin@imm.ac.cn

**Keywords:** T2DM, KK-Ay mice, insulin, BMD, bone structure, osteoblast, osteoclast

## Abstract

Type 2 diabetes mellitus (T2DM) is a chronic disease characterized by hyperglycemia, hyperinsulinemia and complications, including obesity and osteoporosis. Rodents have been widely used to model human T2DM and investigate its effect on the skeleton. We aimed to investigate skeletal alterations in Yellow Kuo Kondo (KK-Ay) diabetic mice displaying high insulin and glucose levels. Bone mineral density (BMD), micro-architecture and bone metabolism-related genes were analyzed. The total femoral areal BMD (aBMD), cortical volumetric BMD (vBMD) and thickness were significantly increased in KK-Ay mice, while the trabecular vBMD and mineralized bone volume/tissue volume (BV/TV), trabecular thickness and number were decreased compared to C57BL mice. The expression of both osteoblast-related genes, such as osteocalcin (*OC*), bone sialoprotein, Type I Collagen, osteonectin, *RUNX2* and *OSX*, and osteoclast-related genes, such as *TRAP* and *TCIRG*, were up-regulated in KK-Ay mice. Correlation analyses showed that serum insulin levels were positively associated with aBMD, cortical vBMD and thickness and negatively associated with trabecular vBMD and micro-architecture. In addition, serum insulin levels were positively related to osteoblast-related and osteoclast-related gene expression. Our data suggest that high insulin levels in KK-Ay diabetic mice may increase cortical bone mass and impair trabecular micro-structure by up-regulating osteoblast-and osteoclast-related gene expression.

## 1. Introduction

Type 2 diabetes mellitus (T2DM) is a chronic metabolic disease characterized by elevated blood glucose levels resulting from impaired glucose metabolism and insulin resistance, which transitions into insulin deficiency over time. Diabetes isassociated with complications [1], such as altered bone metabolism that may lead to osteopenia, an increased risk of fracture and osteoporosis [2]; however, the causal relationship between diabetes and bone loss has been controversial, and the bone diseases that develop in type 1 and type 2 diabetes may differ. A reduction in bone mass in Type 1 diabetes mellitus (T1DM) is generally accepted to be related to high fracture risk resulting from a lack of insulin [3]; however, patients with T2DM often have normal or slightly high bone mineral density (BMD), suggesting impaired bone quality rather than quantity [4,5,6]. The apparent paradoxes suggest that the increased bone fragility in T2DM may not be discerned from bone mass; however, the underlying mechanisms are not completely clear.

Hyperglycemia has been implicated in the pathogenesis of diabetic bone disease. *In vitro*, high glucose significantlyimpairs bone formation by inhibiting osteoblast proliferation and differentiation [7] and suppresses bone resorption [8]. Additionally, high glucose is associated with low BMD [2]. Clinical evidence has shown elevated BMD in patients with T2DM, which is characterized by increased insulin levels in the early phase [9], because insulin promotes osteoblast proliferation, collagen synthesis, and alkaline phosphatase production [10]. In addition, a functional loss of osteoblasts in the presence of high insulin levels is enhanced by increased glucose levels *in vitro* [11]. Although the *in vitro* contribution of glucose and insulin to bone metabolism has been studied, it is unclear whether high glucose or high insulin concentrations affect micro-structures in T2DM animal models.

Various T2DM rodent models have been established that mimic the skeletal characteristics of human T2DM, and these have provided important insights into diabetic fractures. However, most studies on the skeleton have focused on high fat diet-induced obesity, and these models rarely develop diabetes [12]. Yellow Kuo Kondo (KK-Ay) diabetic mice are a classic animal model of T2DM that develop significantly higher glucose and insulin levels compared with the high fat diet-induced obesity model. Thus, KK-Ay mice can serve as an advantageous model for investigating the effect of long-standing high insulin and glucose levels on bone turnover, bone mass and micro-structure. However, very little information is available on the skeleton of KK-Ay mice, with a single study reporting that the proximal femur BMD was lower in KK-Ay mice [2]. Therefore, it is necessary to study the effect of high insulin and glucose levels in KK-Ay diabetic mice on the bone micro-structure.

Thus, we utilized KK-Ay mice to measure the BMD and bone micro-structure. We found high insulin and glucose levels in KK-Ay mice, and that the total femoral aBMD and cortical vBMD were higher, but the trabecular vBMD and micro-structure were impaired. In addition, we also detected the up-regulation of bone metabolism-related genes. These findings suggest that high insulin levels are associated with increased bone metabolism-related gene expression in KK-Ay mice, which subsequently may increase BMD and impair trabecular micro-architecture.

## 2. Results

### 2.1. Serum Insulin, Glucose and Osteocalcin Levels in KK-Ay Mice

**Figure 1:**
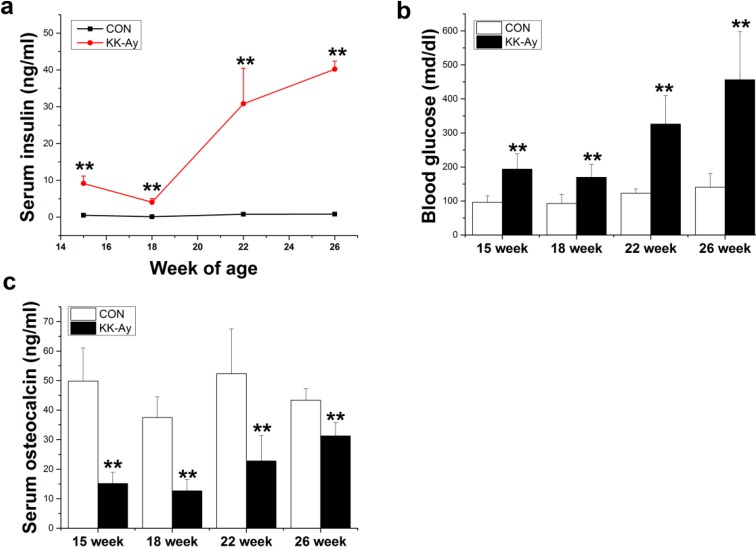
Serum biochemical parameters in control (CON)and KK-Ay mice (**a**) serum insulin; (**b**) fasting blood glucose; (**c**) serum osteocalcin, *n* = 10, ** *p* < 0.01 *vs.* CON.The data are shown as the means ± SE.

Serum insulin levels were significantly higher in KK-Ay mice (9.2 ± 2.02, 4.0 ± 1.02, 30.8 ± 9.66, 47.2 ± 2.17 ng/mL at 15, 18, 22 and 26 weeks, respectively) than in the control group (0.51 ± 0.097, 0.11 ± 0.008, 0.79 ± 0.098, 0.82 ± 0.021 ng/mL at 15, 18, 22 and 26 weeks, respectively) (*p* < 0.01) (Figure 1a). Serum glucose levels of KK-Ay mice were also significantly increased in comparison with the control group (193.7 ± 44.7, 169.4 ± 38.6, 326 ± 83, 456.3 ± 141.9 md/dL *vs.* 96.1 ± 18.75, 92.7 ± 26.6, 122.6 ± 12.3, 140 ± 40.26 md/dL at 15, 18, 22 and 26 weeks, respectively) (*p* < 0.01) (Figure 1b). Additionally, serum osteocalcin concentrations were lower in KK-Ay diabetic mice (15.1 ± 3.8, 12.6 ± 3.9, 22.8 ± 8.5, 31.2 ± 4.5 ng/mL at 15, 18, 22 and 26 weeks, respectively) than in the control group (49.8 ± 11.2, 37.5 ± 6.9, 52.3 ± 15.2, 43.4 ± 3.9 ng/mL at 15, 18, 22 and 26 weeks, respectively) (*p* < 0.01) (Figure 1c).

### 2.2. Areal BMD in KK-Ay Diabetic Mice

**Figure 2:**
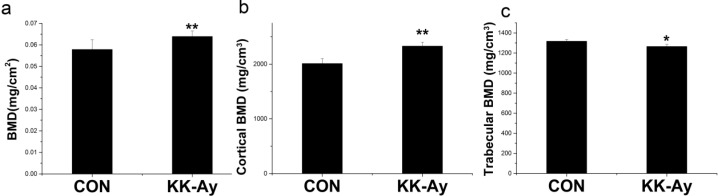
Femoral BMD in control (CON) and KK-Ay mice The areal bone mineral density (aBMD) of the control and KK-Ay mice were measured using dual energy X-ray absorptiometry, and the volumetric BMD (vBMD) was detected using micro-computed tomography measurements. (**a**) BMD of right femur; (**b**) cortical vBMD of right distal femur; (**c**) trabecular vBMD of right distal femur; *n* = 4–10, * *p* < 0.05 *vs.* CON, ** *p* < 0.01 *vs.* CON. The data are shown as the means ± SE.

Dual energy X-ray absorptiometry analyses revealed that the femoral aBMD was significantly higher in KK-Ay mice than in the control group (*p* < 0.01) (Figure 2a).

### 2.3. Volumetric BMD in KK-Ay Diabetic Mice

After the DEXA analysis of the femur, non-destructive μCT was used to evaluate the vBMD in the distal femur. The cortical vBMD (Figure 2b) was significantly higher, but the trabecular vBMD (Figure 2c) was remarkably lower in KK-Ay mice compared with the controls (*p* < 0.05).

### 2.4. Histological Analyses

**Figure 3:**
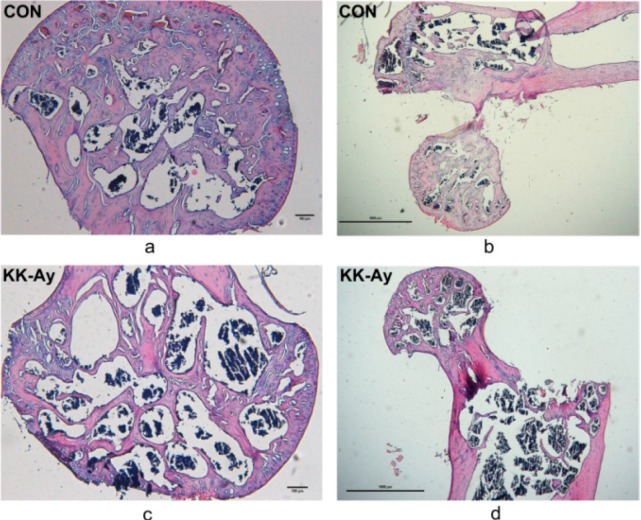
Histology photomicrographs showing H&E staining of the micro-structure of cancellous bone in demineralized paraffin sections. Representative photographs of histological sections from the proximal femora in control (**a**,**b**) and KK-Ay mice (**c**,**d**). Scale bar: 100 μm in (**a**,**c**) and 1000 μm in (**b**,**d**).

H&E staining of sections (Figure 3a–d) demonstrated that the trabeculae were smaller and less connected in the femoral heads of KK-Ay diabetic mice.

### 2.5. Bone Micro-Structure Analyses

**Figure 4:**
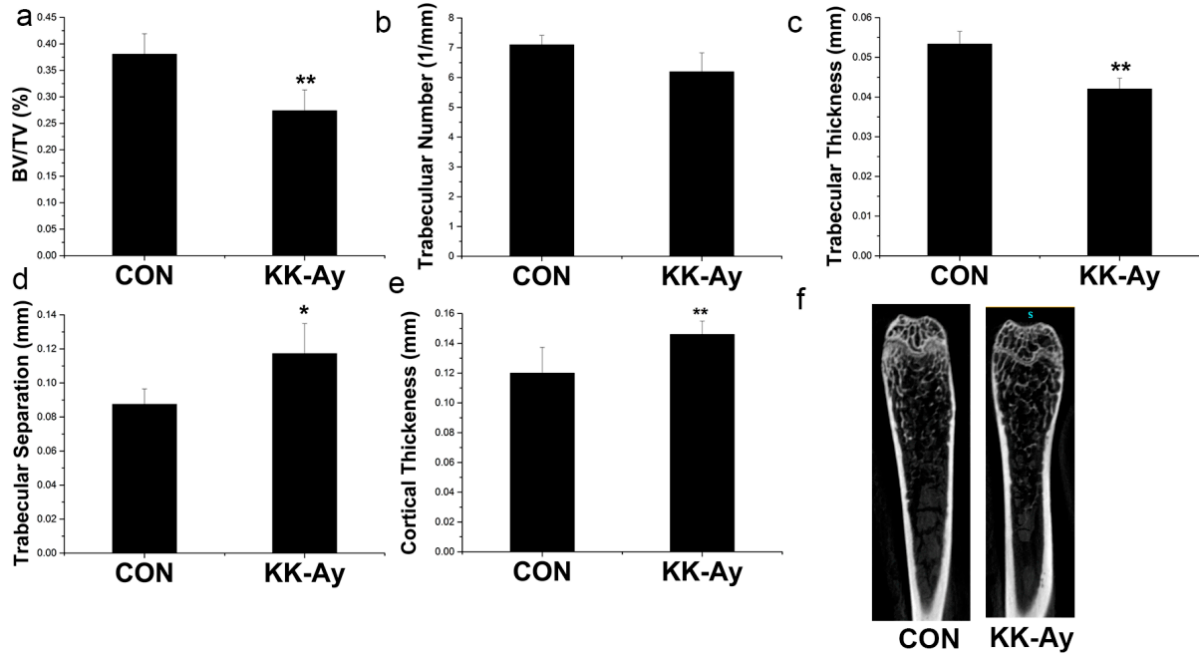
Bone micro-architecture in control (CON) and KK-Ay mice. All parameters are micro-computed tomography derived. (**a**) BV/TV (bone volume/total volume); (**b**) trabecular number; (**c**) trabecular thickness; (**d**) trabecular separation; (**e**) cortical wall thickness; (**f**) µCT images of the distal femur; *n* = 4–5, ** *p* < 0.01 *vs.* CON; * *p* < 0.05 *vs.* CON. The data are shown as the means ± SE.

There was a significant decrease in the BV/TV and trabecular thickness in KK-Ay diabetic mice (*p* < 0.05) (Figure 4a,c). Trabecular numbers showed a trend of reduction in the KK-Ay diabetic mice compared with the control group (Figure 4b); however, trabecular separation was significantly increased in KK-Ay diabetic mice compared with the controls (*p* < 0.05) (Figure 4d). In addition, a higher cortical thickness was observed in KK-Ay diabetic mice compared with the controls (*p* < 0.05) (Figure 4e). μCT images of the distal femur demonstrated that the trabeculae were thinner, smaller, and less connected and the cortical bone was thicker in KK-Ay diabetic mice (Figure 4f).

### 2.6. Bone Metabolism

#### 2.6.1. Enhanced Osteoblast-Related Gene Expression in KK-Ay Diabetic Mice

**Figure 5:**
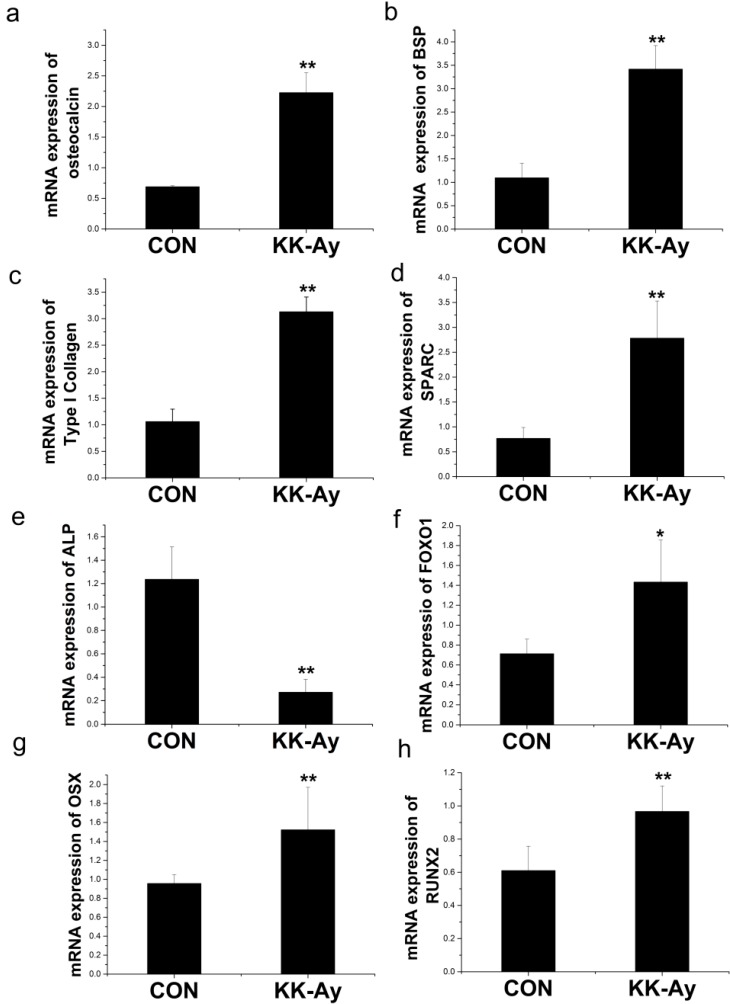
The expression of osteoblast-related genes in control (CON) and KK-Ay diabetic mice RNA was extracted from the bone of control and KK-Ay mice, reverse transcribed, and subjected to real-time PCR analysis. (**a**) mRNA expression of osteocalcin; (**b**) mRNA expression of bone sialoprotein (*BSP*); (**c**) mRNA expression of Type I Collagen; (**d**) mRNA expression of osteonectin; (**e**) mRNA expression of alkaline phosphatase(*ALP*); (**f**) mRNA expression of *FOXO1*; (**g**) mRNA expression of *OSX*; (**h**) mRNA expression of *RUNX2*; *n* = 5–8, ** *p* < 0.01 *vs.* CON; * *p* < 0.05 *vs.* CON. The data are shown as the means ± SE.

To investigate the underlying molecular mechanism for the altered BMD and micro-structure in KK-Ay diabetic mice, osteoblast-related gene expression was assessed. Osteocalcin (*OC*), bone sialoprotein (*BSP*), Type I Collagen, and osteonectin (*SPARC*) expression were increased by two-fold in the KK-Ay diabetic mice (Figure 5a–d), whereas alkaline phosphatase (*ALP*) expression, an early marker of bone formation, was decreased 80% compared to the control group (Figure 5e). The expression of transcription factors *FOXO1*, *RUNX2*, and *OSX* was increased by approximately 30% in the KK-Ay diabetic mice compared to the controls (Figure 5f–h). These results showed that bone formation was enhanced in KK-Ay diabetic mice.

#### 2.6.2. Enhanced Osteoclast-Related Gene Expression in KK-Ay Diabetic Mice

**Figure 6:**
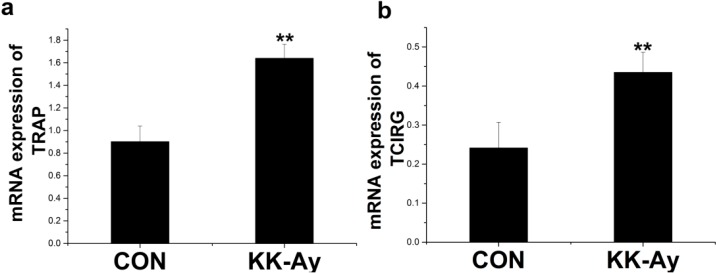
The expression of osteoclast-related genes in control (CON) and KK-Ay mice RNA was extracted from the bone of control and KK-Ay mice, reverse transcribed, and subjected to real-time PCR analysis. (**a**) mRNA expression of TRAP; (**b**) mRNA expression of TCIRG; *n* = 3–5, ** *p* < 0.01 *vs.* CON. The data are shown as the means ± SE.

Osteoclastogenesis-related gene expression, including tartrate-resistant acid phosphatase (*TRAP*) and T cell immune regulator (*TCIRG*), was analyzed. The expression of *TRAP* was increased approximately 1.8-fold (Figure 6a) and the expression of *TCIRG* was increased approximately1.5-fold in KK-Ay diabetic mice (Figure 6b). Therefore, bone resorption was significantly enhanced in KK-Ay diabetic mice.

### 2.7. Correlation Analyses

Serum insulin levels were positively associated with the femoral aBMD, cortical vBMD, and cortical thickness; however, serum insulin levels were negatively correlated with the trabecular vBMD, BV/TV, trabecular number and thickness and positively correlated with trabecular separation (Table 1). Insulin levels were positively related to expression of the osteoclast-related gene *TRAP* (Table 2). A positive relationship was found between serum insulin levels and the expression of osteoblast-related genes, including osteocalcin, *BSP*, Collagen I, *SPARC*, *Foxo1*, *RUNX2*, and *OSX* (Table 2).

**Table 1:** Correlation analysis between serum insulin, glucose levels and bone mass and micro-structure parameters at 26 week.

Parameter	Insulin	Glucose
	*n* = 17	*n* = 17
aBMD of the total femur	0.62 **	0.59 *
Cortical vBMD	0.90 **	
Trabecular vBMD	−0.82 *	
Ct Th	0.75 *	
Tb Th	−0.91 *	−0.73 *
BV/TV	−0.84 **	−0.75 **
Tb N	−0.88 *	
Tb Sp	0.77 *	

** Correlation is significant at the 0.01 level; * Correlation is significant at the 0.05 level. The blank represents no significant correlation. Ct Th, cortical thickness; Tb Th, trabecular thickness; Tb N, trabecular number; Tb Sp, trabecular separation.

**Table 2:** Pearson’s correlation coefficients between serum insulin, glucose levels *vs.* bone turnover markers at 26 week.

Parameter	Serum Insulin ( *n* = 10)	Serum Glucose ( *n* = 10)
Osteoblast-related gene		
Osteocalcin	0.96 **	
*ALP*	−0.94 **	
*BSP*	0.96 **	
Collagen I	0.98 **	
*SPARC*	0.96 **	0.76 *
*FOXO1*	0.82 *	
*RUNX2*	0.80 *	
*OSX*	0.69 *	
Osteoclast-related gene		
*TRAP*	0.97 **	

** Correlation is significant at the 0.01 level; * Correlation is significant at the 0.05 level. The blank represents no significant correlation.

Serum glucose levels were positively associated with the femoral aBMD but negatively correlated with the trabecular BV/TV and thickness (Table 1). Glucose levels were positively associated with expression of the osteoblast-related gene SPARC (Table 2).

## 3. Discussion

Our results demonstrate that KK-Ay diabetic mice display increased insulin and glucose levels from 15 to 26 weeks compared with C57BL male mice. In addition, KK-Ay mice displayed a high areal bone mass and cortical bone mass but a decreased trabecular vBMD and impaired trabecular micro-architecture, which may have reduced bone quality. Furthermore, both osteoblast-related and osteoclast-related gene expression were up-regulated. Correlation analyses showed that serum insulin levels were positively correlated with cortical bone mass and osteoblast- and osteoclast-related gene expression but negatively correlated with trabecular micro-architecture. Therefore, high insulin levels in the KK-Ay diabetic mice are associated with altered osteoblast- and osteoclast-related gene expression, which may increase cortical bone mass and impair trabecular micro-structure.

Growing evidence suggests that patients with T2DM have an increased risk of fracture despite normal to high BMD [2,9,10,11,12]; however, the factors underlying the increased fracture risk in T2DM are poorly understood. Thus, to gain insight into themechanisms contributing to skeletal quality in T2DM, there is a need for relevant animal models.The KK-Ay mouse is a classic, obese T2DM animal model. KK-Ay mice progress through several developmental stages into diabetes when maintained under standard conditions. KK-Ay mice develop from an insulin-resistant stage with hyperinsulinemia and euglycemia to a hyperinsulinemia, hyperglycemic, and insulin-deficient stage [13]. In our study, the development of hyperglycemia and hyperinsulinemia was observed in the KK-Ay mice compared with C57BL male mice, which demonstrated the onset of T2DM pathophysiology.

Bone mass is tightly regulated by osteoclastic and osteoblastic bone remodeling. The contribution of high glucose and insulin to bone mass and bone structure is poorly understood *in vivo* [14]. Cross-sectional studies have examined cortical and trabecular bone density. The most consistent observation is that the trabecular vBMD in T2DM is generally similar to or significantly greater than that of nondiabetic controls [15,16,17]; however, the effects of T2DM on cortical bone are inconsistent, with some studies reporting no differences [16], and others reporting deleterious changes in cortical bone structure in T2DM [17,18]. Recent studies in diet-induced obesity (DIO) models have shown that the effects of a high fat diet (HFD) on bone mass and micro-structure are more variable. For example, some studies have reported that a HFD has beneficial effects on bone mass and trabecular micro-architecture [19,20], and other shave indicated that a HFD causes bone loss and impaired trabecular micro-architecture [21]. Animal studies have mainly focused on the high fat diet-induced obese model, but this model is not ideal for diabetes research because it never develops frank diabetes [12]. In the present study, we used KK-Ay mice, which maintained high insulin and glucose levels throughout the study. We found that the aBMD, cortical vBMD and thickness were higher, but the trabecular vBMD was lower in KK-Ay mice compared with C57BL mice. Moreover, the BV/TV and trabecular thickness and number were reduced, but trabecular separation was increased, suggesting impaired trabecular micro-architecture. Therefore, the high bone mass may have been due to increased cortical bone mass and thickness, but the trabecular micro-architecture was impaired.

Bone is a dynamic organ that undergoes continuous remodeling (bone turnover), which involves bone resorption by osteoclasts followed by bone formation by osteoblasts. Therefore, bone mass is the net product of coordinated bone formation and resorption. Transcription factors such as *Runx2* and *OSX* are involved in regulating the multistep molecular pathway of osteoblast differentiation [22], and *Runx2* is a key downstream regulator of the canonical Wnt/β-catenin pathway, which plays a crucial role in the control of osteoblastogenesis and bone formation [23]. Osteoblast-related genes (osteocalcin, Type I Collagen, *BSP*) promote bone formation, while osteoclast-related genes (*TCIRG*, *TRAP*) play a critical role in bone resorption [24]. Clinically, high BMD is associated with decreased bone resorption and increased bone formation in T2DM [25]. Studies using diet-induced obesity rodent models show high bone mass and consistently observe decreased bone formation markers, such as serum *ALP* activity, and increased bone resorption markers, such as serum carboxy-terminal collagen crosslinks (*CTX*) levels, which mainly result in bone loss and deleterious changes in trabecular architecture [21,26]. In the present study, our results showed that the expression of osteoblast- and osteoclast-related genes was enhanced in KK-Ay diabetic mice. Therefore, elevated bone formation and bone resorption contributed to high aBMD and cortical bone mass. Increased osteoclast-related gene expression, including *TRAP* and *TCIRG* genes, may be responsible for the impaired trabecular vBMD and micro-architecture in KK-Ay diabetic mice, which may result in reduced bone quality.

Insulin acts as an osteo-anabolic agent *in vivo* and *in vitro*. In the present study, correlation analyses showed that insulin levels were positively associated with the cortical vBMD, while they negatively influenced the trabecular vBMD and architecture, specifically the BV/TV and trabecular number and thickness. Additionally, serum glucose levels were positively associated with the femoral aBMD, but were negatively correlated with the trabecular BV/TV and thickness. Correlation analyses indicated that serum insulin levels were positively related to the expression of osteoblast-related genes, such as osteocalcin, *BSP*, Collagen I, *SPARC*, *RUNX2*, and *OSX*, and osteoclast-related genes, including *TRAP*. The results showed that high insulin levels were associated with increased osteoblast-related and osteoclast-related gene expression in KK-Ay diabetic mice. Compared with high glucose levels, high insulin levels had a greater negative influence on the trabecular micro-architecture. Therefore, high insulin levels in KK-Ay diabetic mice may cause increased bone mass and impaired trabecular micro-structure through the up-regulation of bone metabolism-related genes.

## 4. Experiential Section

### 4.1. Animals and Treatment

Eleven-week-old male KK-Ay mice (KK-Ay, *n* = 7) were obtained from the Animal Center of the Institute of Laboratory Animal Sciences, Chinese Academy of Medical Sciences and Peking Union Medical College. C57BL male mice served as the controls (CON, *n* = 10). The animals were housed in a facility maintained at 21–23 °C and a relative humidity of 40%–60%. The animals were exposed to a 12-h light-dark cycle (6:00 a.m.–6:00 p.m. light, 6:00 p.m.–06:00 a.m. dark) and were allowed *ad libitum* access to water and chow for 15 weeks (1K65, Beijing HFK Bioscience Co., Ltd., Beijing, China). All experimental procedures were approved by the Institutional Animal Care and Use Committee of the Institute of Materia Medica, Chinese Academy of Medical Sciences. At 11 weeks of age, non-fasting blood glucose measurements of blood from the lateral saphenous vein were obtained using a glucose analyzer (Biosen 5030, EKF Diagnostic, EKF Diagnostic, Magdeburg, Germany). Mice with blood glucose levels >200 mg/dL were considered diabetic.

### 4.2. Biochemical Analyses

During the experiment, blood samples were collected from mice at 15, 18, 22 and 26 weeks after an overnight fast. Serum osteocalcin (Immutopics, San Clemente, CA, USA) and insulin levels (ALPCO, Windham, NH, USA) were measured using an enzyme-linked immunosorbent assay. The intra- and inter-assay coefficients of variation (CV) were less than 10%.

### 4.3. Bone Mineral Density Analyses

After the mice were sacrificed, the femurs were removed and cleansed of muscles and tendons. The right femur was immersed in a 10% formaldehyde solution and measured by dual energy X-ray absorptiometry (Lunar PIXImus, Madison, WI, USA).

### 4.4. Micro-Computed Tomography Measurements

The influence of T2DM on trabecular and cortical bone mass and micro-structure was assessed at the distal femur metaphysis. The right total femur was scanned using an Inveon MM CT (SIEMENS, Knoxville, TN, USA) at 17 µm isotropic voxel size with an X-ray power source of 60 KV and 220 µA and an integration time of 400 ms. Three-dimensional (3D) reconstruction and quantitative analyses were performed using Inveon Research Workplace software (SIEMENS).

A direct 3D evaluation of the trabecular bone structural parameters was carried out in a region of interest (ROI) that consisted of ~30 slices starting from approximately 0.1 mm distal to the growth plate, constituting 0.5 mm in length. The cancellous bone was separated from the cortical regions by semi-automatically drawn contours. The following 3D parameters were analyzed in the defined ROI: bone volume over total volume (BV/TV%), trabecular number (Tb N 1/mm), trabecular thickness (Tb th, mm), trabecular separation (Tb Sp, mm), and cortical wall thickness (mm). The cortical vBMD and trabecular vBMD was the average density of the segmented fraction starting from approximately 0.1 mm distal to the growth plate, constituting 0.5 mm in length.

### 4.5. Histological Sections and Staining

After the μCT evaluation, the femur was fixed in 4% paraformaldehyde and subsequently decalcified for 5 weeks in 10% EDTA. After dehydration and complete decalcification, the samples were embedded in paraffinwax. Then, 5-mm vertical serial slices were prepared using a microtome (RM 2155, Leica, Bensheim, Germany), and sections were stained with hematoxylin and eosin (HE, Merck, Darmstadt, Germany) for microscopic observation.

### 4.6. RNA Isolation and Quantitative Polymerase Chain Reaction (qPCR) Analyses

The left femurs were frozen in liquid nitrogen and homogenized using a mortar and pestle in liquid nitrogen. Total RNA was isolated from bone samples using TRIZOL reagent (Invitrogen, Carlsbad, CA, USA). For the reverse transcriptase reactions, equal amounts of total RNA (500 ng) were incubated for 3 min at 70 °C and subsequently reverse-transcribed into cDNA for 1 h at 42 °C. Real-time PCR was performed with 2 µL of cDNA in a 25 µL reaction volume using an ABI Gene Amp 5700 Sequence Detection System and SYBR Green PCR Master Mix (Qiagen, Hilden, Germany). The cycling conditions were as follows: incubation at 95 °C for 5 min, 40 cycles of denaturation at 94 °C for 30 s, annealing at 56 °C for 30 s and extension at 72 °C for 30 s. The forward and reverse primer sequences are listed in Table 3. The relative expression levels were normalized to β-actin.

**Table 3:** Primer sequences used for real-time RT–PCR.

Gene	Forward Primer	Reverse Primer
	(Sequence 5'–3')	(Sequence 5'–3')
*Osteocalcin*	TGCAAAGCCCAGCGACTCT	AGTCCATTGTTGAGGTAGCG
*ALP*	TCTCCAGACCCTGCAACCTC	CATCCTGAGCAGACCTGGTC
*BSP*	GAAAATGGAGACGGCGATAG	ACCCGAGAGTGTGGAAAGTG
*Collagen I*	TTGACCCTAACCAAGGATGC	CACCCCTTCTGCGTTGTATT
*SPARC*	ATCCAGAGCTGTGGCACACA	GGAAAGAAACGCCCGAAGA
*TCIRG*	GATCATGGGCTCTATGTTCCG	ACCTGCCCGCTGCACTTCTT
*TRAP*	CAGCAGCCAAGGAGGACTAC	ACATAGCCCACACCGTTCTC
*FOXO1*	AGAGGCTCACCCTGTCGCAGA	GTGAAGGGACAGATTGTGGCGA
*RUNX2*	TCCTGGTCACAATGGGATACC	ATCTCCTGGGTCACCCTTAGG
*OSX*	ACTGGCTAGGTGGTGGTCAG	GGTAGGGAGCTGGGTTAAGG
*β-Actin*	GCTCTTTTCCAGCCTTCCTT	AGGTCTTTACGGATGTCAACG

### 4.7. Statistical Analysis

The data are presented as the means ± standard deviation (SD). The results were evaluated by unpaired Student’s *t*-test or one-way ANOVA using statistical software SPSS (version 13.0). A *p* value <0.05 was considered statistically significant.

## 5. Conclusions

In summary, our results indicate that high insulin levels may be associated with high cortical bone mass and impaired trabecular vBMD and micro-architecture by altering the expression of bone metabolism-related genes.
